# Multi-epitope vaccine against drug-resistant strains of *Mycobacterium tuberculosis*: a proteome-wide subtraction and immunoinformatics approach

**DOI:** 10.5808/gi.23021

**Published:** 2023-09-27

**Authors:** Md Tahsin Khan, Araf Mahmud, Md. Muzahidul Islam, Mst. Sayedatun Nessa Sumaia, Zeaur Rahim, Kamrul Islam, Asif Iqbal

**Affiliations:** 1 Department of Genetic Engineering and Biotechnology, Shahjalal University of Science and Technology, Sylhet 3114, Bangladesh; 2 Infectious Diseases Division, International Centre for Diarrhoeal Disease Research, Bangladesh, Mohakhali, Dhaka, Bangladesh; 3 Department of Biotechnology, Yeungnam University, Gyeongsan 38541, Korea

**Keywords:** immunoinformatics, multi-drug resistance, *Mycobacterium tuberculosis*, subtractive proteomics, vaccine design

## Abstract

*Mycobacterium tuberculosis* (Mtb) is the causative agent of tuberculosis, one of the most deadly infections in humans. The emergence of multidrug-resistant and extensively drug-resistant Mtb strains presents a global challenge. Mtb has shown resistance to many frontline antibiotics, including rifampicin, kanamycin, isoniazid, and capreomycin. The only licensed vaccine, Bacille Calmette-Guerin, does not efficiently protect against adult pulmonary tuberculosis. Therefore, it is urgently necessary to develop new vaccines to prevent infections caused by these strains. We used a subtractive proteomics approach on 23 virulent Mtb strains and identified a conserved membrane protein (MmpL4, NP_214964.1) as both a potential drug target and vaccine candidate. MmpL4 is a non-homologous essential protein in the host and is involved in the pathogen-specific pathway. Furthermore, MmpL4 shows no homology with anti-targets and has limited homology to human gut microflora, potentially reducing the likelihood of adverse effects and cross-reactivity if therapeutics specific to this protein are developed. Subsequently, we constructed a highly soluble, safe, antigenic, and stable multi-subunit vaccine from the MmpL4 protein using immunoinformatics. Molecular dynamics simulations revealed the stability of the vaccine-bound Toll-like receptor-4 complex on a nanosecond scale, and immune simulations indicated strong primary and secondary immune responses in the host. Therefore, our study identifies a new target that could expedite the design of effective therapeutics, and the designed vaccine should be validated. Future directions include an extensive molecular interaction analysis, *in silico* cloning, wet-lab experiments, and evaluation and comparison of the designed candidate as both a DNA vaccine and protein vaccine.

## Introduction

Tuberculosis, an infectious disease caused by the pathogen *Mycobacterium tuberculosis* (Mtb), ranks as the 10th leading cause of death worldwide. The World Health Organization (WHO) reported approximately 1.6 million and 1.4 million deaths in 2018 and 2019, respectively [1,2]. In 2019, two-thirds of global cases were reported in eight countries, with India accounting for the highest percentage (26%), and its neighboring country, Bangladesh, ranking seventh [1]. Tuberculosis has become a significant challenge to medical science due to the diminishing effectiveness of older broad-spectrum antibiotics and instances of treatment failure. These factors have contributed to the development of antituberculosis drug resistance and the spread of multi-drug resistant (MDR) and extensively drug-resistant (XDR) Mtb strains [1-4]. MDR Mtb is characterized by resistance to at least two first-line antibiotics, while XDR Mtb is resistant to at least one quinolone and one of three second-line injectable agents (amikacin, kanamycin, or capreomycin) [5,6]. In 2019, an estimated 78% of MDR tuberculosis was reported among nearly half a million rifampicin-resistant tuberculosis patients [1]. In most cases, effective injectable agents could be administered [7]. Mtb can exhibit either primary (transmitted) resistance or secondary (acquired) resistance [6,8]. Several mechanisms of drug resistance have been studied, and approximately 74 resistance-related genes have been identified [9]. Examples of mutant/resistance-conferring genes include *rpoB* and *ponA1* for rifamycins; *katG*, and *inhA* for isoniazid; *gyrA* and *gyrB* for levofloxacin and moxifloxacin; *Rrs* for amikacin, capreomycin, and kanamycin; *Eis* for kanamycin; and *tlyA* for capreomycin [10,11].

Vaccines minimize the misuse of antibiotics, which in turn helps prevent the spread of antibiotic-resistant bacterial strains [12]. However, broad-spectrum antibiotics may interact with the human microbiome, while vaccines tend to minimally disrupt the microbiota [13]. The bacillus Calmette-Guerin (BCG) vaccine, which is the only licensed vaccine against tuberculosis, exhibits a protective effect for only 10-15 years and its protection gradually wanes with age in adults. As a result, while the BCG vaccine is effective in providing protection against miliary tuberculosis in childhood, its efficacy in protecting against adult pulmonary tuberculosis varies widely, ranging from 0% to 80% [14-17]. Differences in the BCG strain [18], the genetic backgrounds in diverse populations [19], geography [20], and immunization strategies [21] all play crucial roles in the varying efficacy of BCG vaccines. Furthermore, complications associated with the BCG vaccine are more common in immunocompromised patients [22-24]. This failure contributes to the global disease burden of adult pulmonary tuberculosis. Currently, 14 vaccine candidates are undergoing clinical trials [25-27], and several vaccine development studies are targeting 48 Mtb genes (http://www.violinet.org/). Despite these efforts, scientists are still struggling to achieve satisfactory results. Therefore, the development of safe and effective vaccines is urgently needed to address the antibiotic resistance crisis and combat MDR and XDR Mtb strains.

Advances in bioinformatics tools have expedited the process of vaccine development. These computational tools are regularly used in various methods to analyze the published genomes and proteomes of pathogens, with the aim of identifying new vaccine candidates [28,29]. We have used subtractive proteomics to identify potential therapeutic targets against *Brucella abortus* strain 2308 [30] and *Bartonella bacilliformis* [31]. Moreover, we have employed an immunoinformatics approach to design vaccine candidates against severe acute respiratory syndrome coronavirus 2 [32] and West Nile virus [33]. The detailed protocols of vaccine development through the immunoinformatics approach are well established [34-37]. Pre-clinical studies (both *in vivo* and *in vitro*) of the vaccines developed through the immunoinformatics approach against *Chlamydia trachomatis* [38,39], *Streptococcus agalactiae* [40], *Rickettsia prowazekii* [41], viruses (Epstein–Barr virus [42], Crimean-Congo hemorrhagic fever virus [43]), and protozoan parasite (*Toxoplasma gondii* [44]) have proven the efficacy of the resulting vaccines.

In this study, we used subtractive proteomics with various parameters to identify new vaccine targets from the reference strain *M. tuberculosis* H37Rv, and performed a conservancy analysis in 22 other virulent MDR and XDR Mtb strains. Given that more vaccines have been licensed in the last 40 years than new antibiotics [13], we emphasized vaccine development for short-listed targets. We augmented our study by using the immunoinformatics approach to design an immunogenic recombinant subunit vaccine against Mtb strains, which would stimulate both innate and adaptive immune responses [45-48]. Our results identified the MmpL4 (mycobacterial membrane protein Large) protein as a potential therapeutic target. Mtb contains 13 MmpL proteins that export bulky, hydrophobic substrates to support the ability to infect and persist in the host [49, 50]. Among these 13 MmpL proteins, MmpL4 plays a role in the transport of iron/siderophore (carboxymycobactin). This siderophore exportation and iron importation are facilitated by the MmpL4 protein, which interacts with MmpL5 and the periplasmic adaptor proteins MmpS4/5 (mycobacterial membrane protein-small) [51]. Iron is an essential nutrient for many cellular processes, and Mtb produces siderophores to capture iron from hosts. Disruption of proper carboxymycobactin exportation has a detrimental effect on Mtb survival and growth *in vivo* and *in vitro* [52]. MmpL4 is a virulence-conferring protein essential for maintaining normal growth in the murine tuberculosis model. Mutational disruption in MmpL4 causes impaired growth of the Mtb strain and renders it avirulent [53]. Although the MmpL4 protein is part of the RND (resistance, nodulation, and cell division proteins) family, it does not contribute to intrinsic drug resistance or antituberculosis drug resistance, making the MmpL4 protein a promising antituberculosis drug target [52,53]. Furthermore, iron restriction is a common strategy to combat pathogenic infections [49]. Therapeutics designed to disrupt or block the interaction of the MmpL4 protein with MmpL5 and MmpS4/5 proteins could lead to iron deficiency and subsequent eradication of Mtb infection in the host. Given that Mtb has already developed resistance against many antibiotics and evolved into MDR and XDR Mtb, targeting a protein that has not been exposed to any therapeutics would be an ideal candidate to treat MDR and XDR Mtb cases. Currently, there is no drug or antibiotic available that targets the MmpL4 protein. Considering these issues, we believe that MmpL4 would be a suitable candidate for designing therapeutics to combat MDR and XDR cases. We designed an epitope-based subunit vaccine against the MmpL4 protein to combat antimicrobial resistance. However, the designed vaccine requires further wet-lab evaluation and validation to prove its efficacy *in vivo* and *in vitro*. Our future goals for this study include the wet-lab expression and evaluation of the designed vaccine as a DNA vaccine and protein vaccine [39,43].

## Methods

### Subtractive proteomics approach

**Fig. 1.**
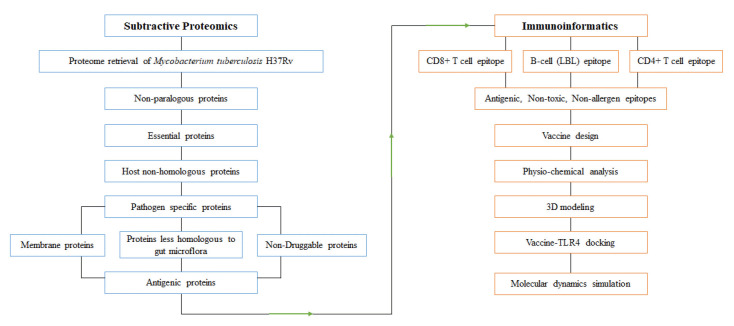
Overall protocol of subtractive proteomics and immunoinformatics approaches. Arrows indicate findings of subtractive proteomics are analyzed in immunoinformatics approach to design potential vaccine candidate.

The subtractive genomic approach was employed to identify novel drug targets and vaccine candidates for the entire proteome of *M. tuberculosis* H37Rv (a representative strain of Mtb). The overall workflow is illustrated in Fig. 1.

### Retrieval of the complete proteome

We retrieved the entire proteome of *M. tuberculosis* H37Rv (Assembly GCA_000195955.2) [54] from the NCBI Genome database. We selected this particular genome since it is recognized as a reference proteome for Mtb.

### Removal of paralogous sequences

The CD-Hit suite [55] was used to identify paralogous or duplicate sequences. The threshold was kept at 0.8 (80% identity) to avoid protein redundancy [56]. Moreover, proteins composed of fewer than 100 amino acids were excluded from the paralogous sequences [57].

### Screening of essential proteins

The essential proteins of *M. tuberculosis* H37Rv were predicted through the Database of Essential Gene (DEG) server v15.2 [58], which contains 53,885 essential genes and 786 essential non-coding sequences. This server is broadly used in the subtractive genomics/proteomics approach to identify essential proteins in organisms [59-61]. The protein hit settings of an expectation value (E-value) ≤ 10^-100^, identity ≥ 25%, and bit score > 100 were used to filter essential proteins for this pathogen [57,62].

### Removal of human homologous proteins

The essential proteins of the pathogen were subjected to BLASTp against the human (host) RefSeq proteome in the Ensemble Genome Database [63]. Any proteins that were identified as host homologous were removed if a significant hit above the threshold value of 10^-4^ was detected [64,65].

### Metabolic pathway analysis

A comparison was conducted between the host and pathogen to identify pathogen-specific pathways (herein termed “unique pathways”) by using the three-letter organism codes “has” and “mtu” for *Homo sapiens* (host) and *M. tuberculosis* H37Rv, respectively, through the Kyoto Encyclopedia of Genes and Genomes (KEGG) Pathway Database [66].

The identified host non-homologous proteins of *M. tuberculosis* H37Rv were subjected to BLASTp through the KAAS server [67] in the KEGG database. Proteins that were involved in unique pathways were identified as unique proteins. Those proteins that were assigned KO (KEGG Orthology) but did not participate in common host-pathogen pathways were also classified as unique proteins. Proteins that were involved in common pathways, as well as those without KO assignments, were excluded from the selection. Hypothetical unique proteins were also omitted from this study.

### Subcellular localization, analysis of druggability, and gut microflora similarity

*M. tuberculosis* H37Rv is a Gram-positive bacterium. As such, we can predict the subcellular localization of previously selected unique proteins—which were non-homologous to the host and specific to the pathogen, and essential—to be extracellular, cell wall, membrane, or cytoplasmic. To ensure the highest accuracy in our predictions, we utilized CELLO v.2.5 [68], PSORTb v3.0.2 [69] and PSLPred [70]. If a particular protein's location was predicted to be the same by either all three or any two of these servers, we adopted that location for our druggability analysis. However, if each server predicted a different location for a specific protein, we excluded that protein from our study.

Unique proteins were screened using the DrugBank v5.1.4 database [71] to assess their novelty as drug targets. The DrugBank Database includes numerous FDA-approved drugs and displays protein targets along with their respective drug IDs. A BLASTp analysis was conducted using default parameters, and proteins that did not match any drug were chosen for further investigation as potential novel drug targets for *M. tuberculosis* H37Rv [30]. Additionally, the molecular weight of these unique proteins was calculated using the ExPASy/ProtParam server [72].

A list of 83 organisms found in the gut flora of healthy humans was compiled from the existing literature (Supplementary File 1) [73]. An NCBI BLASTp analysis was conducted on these unique proteins, with parameters set to an E-value < 0.001, sequence similarity of < 35%, and < 10 hits. The results were then cross-referenced with another set of gut microflora data [74] that contained 95 organisms (Supplementary File 2). Unique proteins that showed <10 hits and <35% similarity were selected for further analysis.

Finally, unique proteins that had a precise location, were predicted as novel drug targets, had a molecular weight <110 kDa, and exhibited low similarity with human gut microflora were selected for an analysis of human anti-targets.

### Human anti-target analysis

The term "anti-target" is used to refer to essential human proteins. In total, 210 anti-targets reported in the literature with their accession numbers (Supplementary File 3) [73] were obtained from the NCBI Protein Database. Previously selected novel drug targets were subjected to BLASTp in NCBI analysis, setting the parameters as an E-value ≤10^–4^, identity <25%, and query length >30%. Only membrane proteins were selected for antigenicity analysis.

### Identification of potential vaccine candidates

Membrane proteins were screened using the VaxiJen v2.0 server [75] and TMHMM Server v. 2.0 [76] with the default parameters. Membrane proteins showing an antigenicity score >0.4 and containing transmembrane helices were selected as vaccine candidates.

### Conservancy analysis

To determine whether the selected proteins were preserved in other virulent Mtb strains, we subjected both cytoplasmic and membrane vaccine targets to BLASTp in NCBI, comparing them against 22 virulent Mtb strains documented in the literature [77,78]. The following cut-off parameters were used to screen proteins: an E-value <0.0001, query cover >80%, and similarity >90%. We selected proteins that passed the screening for protein-protein interaction (PPI) analysis.

### PPI analysis

Proteins form complex interaction networks to function properly. The analysis of these PPIs aids in understanding biological pathways and the structural organization of cells [79]. The most promising metabolic functional associations were identified for selected highly conserved proteins through these interactions. Using STRING v11.0 [80], PPIs for selected proteins were generated with high confidence (>0.700). Additionally, the accession number, gene name, and functions of these interacting proteins were curated from the UniProt database [81].

### Immunoinformatics approach

The design of chimeric subunit vaccines using immunoinformatics has been extensively reviewed in recent years [34,82]. We chose the membrane protein with the highest antigenicity for epitope prediction, which was then used in the construction of the subunit vaccine.

### T-cell epitope prediction

A pool of 9-mer cytotoxic T lymphocyte (CTL; CD8+) epitopes was created using the NetCTL1.2 server [83], with restrictions for 12 human MHC class I supertypes and default parameters. A pool of 15-mer helper T lymphocyte (HTL; CD4+) epitopes was generated using the IEDB MHC II server [84]. The HTL epitopes were chosen based on mouse alleles with a percentile rank of less than 10 and an IC_50_ value of less than 500 nM.

### Linear B-cell epitope prediction

The ABCpred server [85] was used to generate a pool of 20-mer linear B-cell lymphocyte (BCL) epitopes. These epitopes were then cross-checked with the IEDB BepiPred 2.0 prediction method [86].

### Antigenicity, topology, allergenicity, and toxicity predictions of epitopes

All short-listed T-cell and B-cell epitopes were screened using the VaxiJen v 2.0 server to check their antigenicity score. The TMHMM v.20 server was used to predict topology, whereas the AllerTop server [87] and ToxinPred server [88] were used to check the allergenicity and toxicity of these epitopes, respectively. Additionally, the immunogenicity of CTL epitopes was evaluated through the IEDB MHC-I Immunogenicity server [36], and the IFNepitope sever [89] was used to predict interferon-γ (IFN-γ) inducing HTL epitopes.

### Population coverage analysis

Short-listed T-cell epitopes were analyzed using the IEDB Population Coverage tool [90] to predict worldwide coverage. Subsequently, the HLA alleles and their corresponding T-cell epitopes were uploaded to the server.

### Vaccine design

Potential CTL, HTL, and BCL epitopes were combined to construct a subunit vaccine. An AAY linker was utilized to separate CTL epitopes, while the GPGPG linker and KK linker were employed to attach the HTL and BCL epitopes, respectively [91]. In addition, an EAAAK linker separated the L7/L12 Ribosomal protein, a Toll-like receptor-4 (TLR-4) agonist [92], from the universal PADRE sequence at the N-terminal end of the vaccine [93]. An EGGE linker joined the invasin peptide with another PADRE sequence at the C-terminal end of the vaccine [94].

### Physiochemical properties of the designed vaccine and structure construction

The ExPASy-ProtParam server was used to predict several physiochemical properties of the vaccine construct, including molecular weight, theoretical PI, instability index (II), half-life, and aliphatic index (AI). Furthermore, the VaxiJen server was utilized to predict the antigenicity of the constructed vaccine. The AlgPred server [95] and SOLpro tool [96] were employed to predict the allergenicity and protein solubility for the *Escherichia coli* expression system.

The Robetta server [96] was used to predict the three-dimensional (3D) structure of the constructed vaccine. This structure was then refined using the GalaxyRefine server [97]. Next, only the best-refined model was evaluated through the PROCHECK [98] and Verify3D [99] algorithms on the SAVES server and compared with the initially modeled structure. The ProSA webserver [100] was used to predict the Z-score. Finally, a refined structure was selected for docking with human TLR-4.

### Molecular docking, molecular dynamics simulation, and immune simulation

The PDB structure of human TLR-4 (PDB id: 4G8A) was prepared using the SWISSPDB viewer for docking purposes. The ClusPro 2.0 server [101] was utilized to perform molecular docking of the refined vaccine protein with the TLR-4 monomer. The docked complex with the lowest binding energy was then subjected to molecular dynamics (MD) simulation in YASARA [102], utilizing the AMBER14 force field. Initially, the system was cleaned and optimized. The TIP3P model was implemented, with Na/Cl ions added at a density of 1.012 g/cm^3^. The particle mesh Ewald method was used to calculate long-range electrostatic interactions at a distance of 8 Å. The physiological system of the complex was set at 298K with a pH of 7.0 and 0.9% NaCl [103]. A cubic simulation cell was created (126.4688 Å × 126.4688 Å × 126.4688 Å), and the periodic boundary condition was maintained. The MD simulation was then run with a time step of 2.50 fs [103]. Finally, the root mean square deviation (RMSD), root mean square fluctuation (RMSF), the radius of gyration (Rg), and solvent accessible surface area (SASA) were calculated from the trajectory. Non-bonding interaction analysis was carried out using the PDBsum server [104], which reports the number of non-bonded interactions, hydrogen bonds, salt bridges, disulfide bonds, and their respective contact residues. An immune simulation analysis was conducted using the C-ImmSim server to evaluate the primary and secondary immune responses to the designed vaccine [104]. The doses were administered at intervals of 1, 84, and 168 time steps, with each time step equivalent to 8 hours [105].

## Results

### Retrieval of the complete proteome

The reference strain *M. tuberculosis* H37Rv, retrieved from the NCBI Genome Browser, contains 3906 proteins. These proteins were short-listed through screening with different web-based bioinformatics servers, and the results are shown in Table 1.

### Removal of paralogous sequences

The CD-Hit suite identified 35 paralogous clusters (with a similarity greater than 80%) within the entire proteome of the pathogen. It also detected a total of 46 redundant or duplicate sequences, which could potentially lead to false-positive results in subsequent analyses. Consequently, these duplicate sequences were omitted from further examination [30]. Proteins larger than 100 amino acids are more likely to play a role in the essential metabolic pathways of the organism than smaller proteins (those less than 100 amino acids in length). Therefore, even though they are unique to the organism, a total of 292 protein sequences were excluded on the assumption that they were unlikely to be essential proteins [62].

### Screening of essential proteins

The most critical criterion for a therapeutic target is its potential indispensability for the pathogen's survival [106]. Out of 3565 non-paralogous sequences of the pathogen, only 580 proteins met the screening criteria (E-value ≤10^-100^, identity ≥25%, bit score >100) through the DEG v15.2 server. Therefore, these 580 proteins were ultimately identified as essential proteins.

### Removal of human homologous proteins

Therapeutics targeting proteins from the pathogen that are homologous to those in the host (*H. sapiens*) could potentially disrupt host metabolism and trigger cytotoxic reactions. However, out of the total, 250 essential proteins demonstrated hits above the threshold (E-value >10^-4^) with the RefSeq proteome of humans. Consequently, these proteins, believed to have host homologs, were omitted from our analysis. This exclusion reduces the likelihood of cross-reactivity when a therapeutic is administered to fight the pathogen.

### Metabolic pathway analysis

The metabolic pathways of *M. tuberculosis* H37Rv were analyzed in comparison to human metabolic pathways using the KEGG server. Both the host (“has”) and the pathogen (“mtu”) metabolic pathways were curated from the KEGG Pathway Database. This comparison was conducted to identify shared pathways and those specific to the pathogen. Essential proteins from the host that were non-homologous were subjected to BLASTp analysis via the KAAS server at KEGG. Out of 330 proteins, 117 were found to be involved in shared pathways. A total of 166 proteins were not assigned a KO, suggesting that they are not metabolic proteins. Consequently, these 28 proteins were also excluded from our study. The remaining 47 proteins were assigned to the KO, indicating their involvement in pathogen-specific pathways. However, six proteins were hypothetical and therefore excluded from the study (Supplementary Table 1). As a result, 41 proteins associated with pathogen-specific pathways were short-listed for further investigation [73,107].

### Subcellular localization, analysis of drug target novelty, and gut microflora similarity

Advances in bioinformatics, cheminformatics, and genome sequencing, when combined with experimental data, have shown that several key factors contribute to the identification of potential drug targets in pathogenic organisms. These factors include the prediction of subcellular localization, the novelty of the drug target, molecular mass (kDa), and the presence of transmembrane helices [108-110].

Cytoplasmic proteins serve as suitable drug targets, while membrane proteins are viewed as potential drug targets and vaccine candidates [59]. A short list of 41 proteins from the Gram-positive bacterium *M. tuberculosis* H37Rv was subjected to CELLO, PSORTb, and PSLPred to predict subcellular localization with improved accuracy. Out of the 41 proteins, 23 were predicted to be cytoplasmic proteins, 16 were predicted to be membrane proteins, and one was predicted to be an extracellular protein. However, all three servers were unable to accurately predict the location of the remaining protein.

The primary objective of this study was to identify new vaccine targets for MDR and XDR Mtb strains. To this end, we screened 41 pre-selected proteins through the DrugBank Database to determine their potential novelty as drug targets. Of these 41 proteins, 11 demonstrated significant homology with drug targets of other pathogenic strains. Despite the administration of antibiotics to eliminate other pathogenic bacteria, there is a possibility that these 11 proteins may be exposed to antibiotics or antibacterial drugs. Consequently, the remaining 30 proteins, which did not match any known drug targets, were deemed to be novel drug targets.

The 41 short-listed proteins were screened using NCBI BLASTp to minimize the risk of inadvertently inhibiting the gut microflora present in a healthy human. These gut prokaryotic symbionts play a crucial role in assimilating poorly digestible dietary components, degrading xenobiotics, synthesizing vitamins, and providing resistance against colonization by opportunistic bacteria and other pathogens [111-114]. Consequently, any deterioration of these gut microflora strains could result in nutritional deficiencies in the host and a weakened first-line defense against pathogen invasion [112]. Initially, the 41 short-listed proteins were compared with 83 organisms cited in the literature, using parameters set at an E-value of <0.001, sequence similarity of <35%, and <10 hits. The results were then cross-verified with a set of 95 organisms mentioned in other literature. Ultimately, only nine proteins successfully passed the gut microflora non-homology screening.

It is feasible to experimentally study proteins with a molecular mass of less than 110 kDa. However, proteins with a molecular mass greater than 110 kDa are not suitable as therapeutic targets due to their larger structures [59,109]. Therefore, we determined the molecular weight of the 41 short-listed unique proteins using the ExPASy/ProtParam server.

Finally, five membrane proteins were chosen based on their minimal similarity to human gut microflora, the novelty of their drug target, and a molecular weight of less than 110 kDa. The screening data can be found in Table 2.

### Human anti-target analysis

The removal of unique proteins that are homologous to human anti-targets is crucial in the identification of an appropriate therapeutic target. Human 'anti-targets' encompass the ether-a-go-go related gene (hERG), the constitutive androstane receptor, the pregnane X receptor, P-glycoprotein, and certain membrane receptors such as muscarinic M1, adrenergic a1a, serotonergic 5-HT2C, and dopaminergic D2 [115]. Between 1960 and 1999, several drugs were withdrawn due to adverse reactions with human anti-targets [116], such as benoxaprofen in 1982 [117] and trovafloxacin in 1999 [118]. Therefore, the five selected proteins were subjected to BLASTp on the NCBI server, with parameters set to an E-value ≤10±, identity <25%, and query length >30%. Fortunately, all five proteins successfully passed the screening, thereby reducing the potential for adverse side effects when therapeutics are administered to humans.

### Identification of potential vaccine candidates

Reverse vaccinology has been demonstrated to be a practical approach for discovering vaccine candidates [119]. Emerging safe recombinant vaccines, based on antigenic protein sequences, are becoming the most appealing and cost-effective solution in the battle against infectious diseases. An ideal antigenic protein should have an antigenicity score greater than 0.4, as determined by the VaxiJen server, and should possess transmembrane helices [120]. Of the five proteins examined, all had transmembrane helices, but only one protein, NP_214964.1 (transmembrane transport protein MmpL4), exhibited antigenicity with a VaxiJen score exceeding 0.4 (Supplementary Table 2). Consequently, this MmpL4 protein (‘NP_214964.1’) has been selected as a potential vaccine candidate, in addition to being considered a novel drug target.

### Conservancy analysis

A list of 22 other virulent MDR and XDR Mtb strains has been reported in the literature (Supplementary Table 3) [77,78]. One membrane vaccine candidate (transmembrane transport protein MmpL4, NP_214964.1) of *M. tuberculosis* H37Rv was subjected to BLASTp on the NCBI server (E-value <0.0001, query coverage >80%, sequence similarity >90%) against these 22 MDR and XDR Mtb strains. This protein was found to be present in all 22 virulent strains with at least 99% similarity. Therefore, infections caused by these 23 strains in humans could potentially be treated if therapeutics are designed against any of these essential, unique proteins of Mtb.

To the best of our knowledge, this protein has not been mentioned as a potential novel therapeutic target in previous research [77,78,121-124]. Our research presents a new target for the development of efficacious treatments against infections resulting from MDR and XDR Mtb strains.

### PPI analysis

**Fig. 2.**
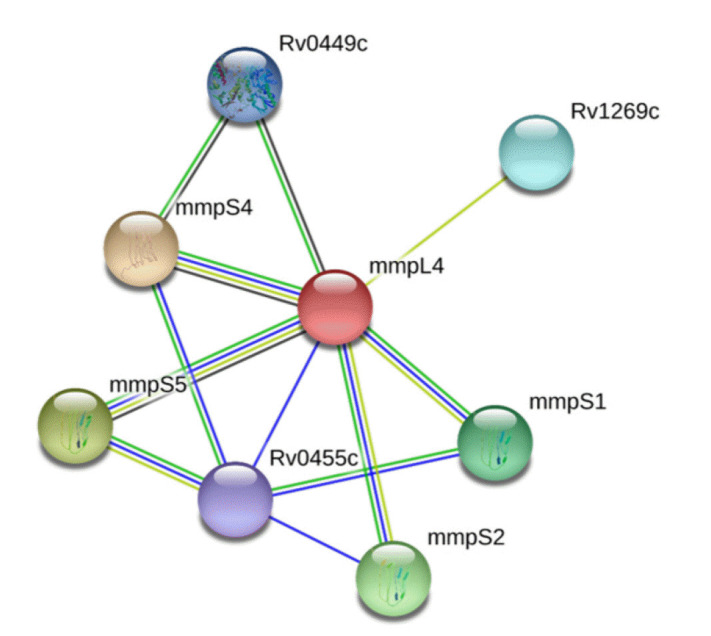
Protein-protein interaction analysis of ‘MmpL4’ protein. ‘MmpL4’ interacted with seven other proteins and among them two proteins ‘MmpS4’ and ‘MmpS5’ were involved in pathogenesis.

PPIs, which are essential for proper functioning, take place in the cellular network, biological pathways, and structural organization of a cell. One of the primary challenges in bioinformatics is the computational analysis of these intricate networks, which aids in understanding whole-cell engineering, drug targets, and cellular activities [79]. The STRING database, with a high confidence score of 0.700, indicates that NP_214964.1 interacts with seven proteins (Fig. 2). Any disruption of these interactions is expected to affect several vital pathways of the pathogen.

### T-cell epitope generation

NetCTL predicted a total of 245 epitopes, which are restricted for MHC class I human super types such as A1, A2, A3, A24, A26, B7, B8, B27, B39, B44, B58, B62. Out of these, 37 epitopes were found to be antigenic, immunogenic, non-toxic, and non-allergic, with an external topology. These characteristics are crucial for CTL epitope selection [125]. Ultimately, four peptide regions containing epitope clusters (^23^MIHAFAVPIILGW^33^, ^221^SIITVVLLLITVGVEL^236^, ^380^VVVRWPLPVLV^360^, and ^514^SVLLWQHILAIHLHWL^529^) were selected for vaccine construction (Supplementary Table 4).

In contrast to CTL, the IEDB MHC II server predicted 50 epitopes restricted for mouse H-2-I alleles. Of these, 18 epitopes were predicted to be antigenic, non-toxic, and non-allergic with an external topology. Only 12 HTL epitopes were found to be capable of inducing IFN-γ secretion (Supplementary Table 5). Finally, three peptide regions containing epitope clusters (^377^VGTVVVRWPLPVLVA^391^, ^878^NAGLVFAVTMASMAVSDL^895^, and ^925^AALLGRWFWWPLRVRSRP^943^) were selected for vaccine construction.

### B-cell epitope selection

A total of 81 BCL epitopes (20-mer) were predicted using the ABCpred server. However, only 11 of these epitopes demonstrated antigenicity, non-toxicity, and non-allergenicity. These selected epitopes were then cross-verified using the IEDB BepiPred 2.0 method. Ultimately, five peptide regions containing epitope clusters (^49^LEAVGQERSVSLSPKDAPSF^68^, ^181^YVTGPSALAADMHHSGDRSM^200^, ^481^RPEGTTMDHTSIPFQISMQNAGQLQTIKYQ^520^, ^531^LTRMHSLMAEMASTTHRMVGDTEEMKE^557^, and ^932^FWWPLRVRSRPARTPTVPSE^951^) epitopes were selected for vaccine construction (Supplementary Table 6).

### Population coverage analysis of MHC class I and class II restricted epitopes

**Fig. 3.**
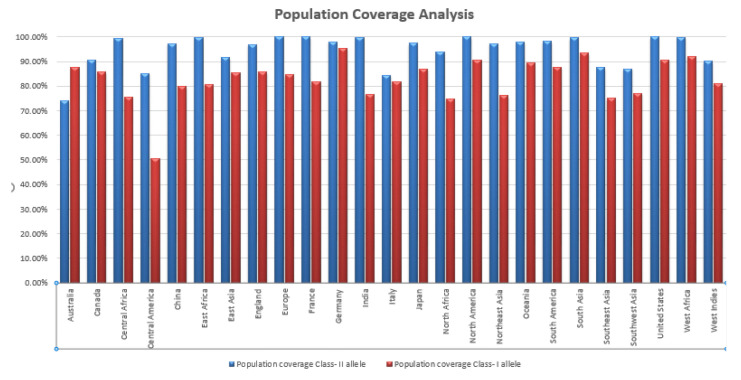
Worldwide population coverage analysis of selected cytotoxic T lymphocyte (CTL) and helper T lymphocyte (HTL) epitopes. CTL epitopes covered 98.84% MHC class I type HLA alleles worldwide whereas HTL covered 86.76% MHC class II type HLA alleles.

Population coverage was determined for both CD8+ T cells and CD4+ T cells, along with their respective HLA alleles. The highest coverage for CTL was reported in North America and the United States (100%), while the highest for HTL was found in Germany (93%). Therefore, approximately 98.84% of the global population could be covered through the selected CTL epitopes, and around 86.76% through the selected HTL epitopes. These results are shown in Fig. 3.

### Vaccine construction and structure generation

**Fig. 4.**
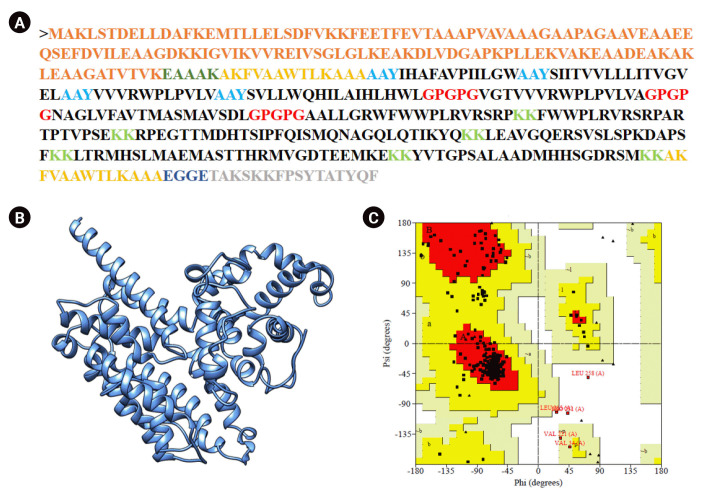
Vaccine construction, 3D structure design and refinement. (A) Selected epitopes were linearly joined with adjuvant (orange color), PADRE sequence (yellow color) and invasin peptide (ash color). Linker were used to separate each other. (B) A refined 3D structure of vaccine containing 443 amino acids. (C) PROCHECK tool revealed that 88.8% residues were present in most favored region and overall 99.5% residues were in allowed region.

A vaccine composed of 443 amino acids was engineered by combining selected CTL, HTL, and BCL epitopes. The 50s ribosomal protein L7/L12 from Mtb (130 amino acids) was attached at the N-terminal end to serve as an adjuvant, stimulating TLR-4. An empirical α-helical linker (EAAAK) was employed to connect the adjuvant with the PADRE (AKFVAAWTLKAAA) sequence. The AAY, GPGPG, and KK linkers were respectively inserted between the CTL, HTL, and LBL. The EGGE linker was utilized to connect the invasin peptide (TAKSKKFPSYTATYQF) with the LBL epitope at the C-terminal end (Fig. 4A).

### Physiochemical analysis of the designed vaccine

The physiochemical properties of the constructed vaccine indicate that it is highly stable (II 36.14), with an estimated half-life of 30 hours in mammalian reticulocytes (*in vitro*). The vaccine is also expected to be thermostable (AI 93.70). Moreover, the vaccine protein is safe, antigenic, and likely soluble in the *E. coli* expression system. The results for all parameters are detailed in Table 3. Three tertiary structures were created using the Robetta server and the *ab initio* modeling algorithm, as no suitable template was available for homology modeling. Out of five models, model 3 had 87.5% of residues in the most favored region. The GalaxyRefine server refined model 3, generating five models, with the first one (Fig. 4B) being the best refined. Furthermore, the PROCHECK server demonstrated that the refined model was an improvement over the initial model 3, with 88.8% of residues in the most favored region (Fig. 4C). The refined model deviated from its initial design by an RMSD of 0.312 Å. The Verify3D and Z-score of the refined model were 89.03 and –6.33, respectively. The parameters of the refined models can be found in Supplementary Table 7.

### Molecular docking, MD, non-bonded interaction analysis, and immune simulation

**Fig. 5.**
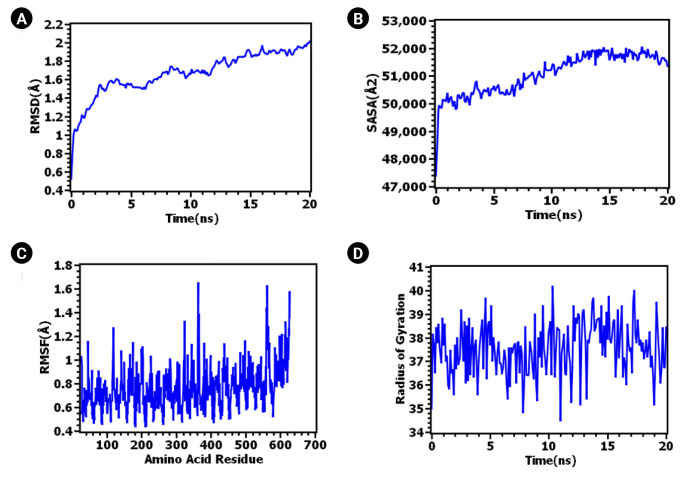
Vaccine construction, 3D structure design and refinement. (A) Selected epitopes were linearly joined with adjuvant (orange color), PADRE sequence (yellow color) and invasin peptide (ash color). Linker were used to separate each other. (B) A refined 3D structure of vaccine containing 443 amino acids. (C) PROCHECK tool revealed that 88.8% residues were present in most favored region and overall 99.5% residues were in allowed region. RMSD, root mean square deviation; SASA, solvent accessible surface area; RMSF, root mean square fluctuation.

The ClusPro 2.0 server generated 30 complexes of TLR-4 bound to the vaccine. Among these, cluster 04 emerged as the best-docked complex, exhibiting the lowest binding energy (-1,114.00 J/Mol). Numerous studies have highlighted the roles of TLR-4 in immunosurveillance and the eradication of the intracellular bacterial pathogen Mtb [126-129]. TLR4 is expressed on both cytoplasmic and endosome membranes and signals through TRIF- and MYD88-dependent pathways [130,131], thereby facilitating immunosurveillance against tuberculosis [132]. To assess the stability of the TLR-4 complex bound to the vaccine, a 20 ns molecular dynamic simulation was conducted. As can be seen in Fig. 5A, the RMSD values increased during the initial phase of the simulation. However, after 10 ns, the RMSD peak appeared to stabilize, maintaining its integrity throughout the 20 ns simulation time. The RMSD values of the systems remained well below 2.5Ǻ for the entire simulation duration. The RMSD of the protein or protein complex signifies the overall stable nature of the protein. Fig. 5B illustrates the rigid nature of the epitope and protein complex [133].

**Fig. 6.**
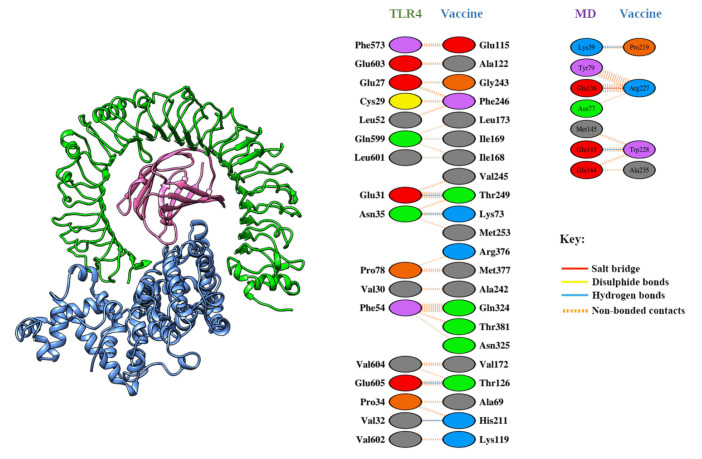
Protein-protein interactions of vaccine bound human Toll-like receptor-4 (TLR-4)/molecular dynamics (MD) complex. Vaccine molecule forms four hydrogen bonds with TLR-4 whereas two hydrogen bonds and one salt bridge interaction with myeloid differentiation factor-2. Rest of the contacts were non-bonded interactions.

The RMSF also provides insight into the flexible regions of the protein [134]. Fig. 5C illustrates the consistent nature of the epitope and protein complex. No excessive fluctuations were noted, although certain amino acids such as Gly363, Lys560, Gln562, Lys615, and Thr626 exhibited higher RMSF compared to other compounds. Conversely, the degree of compactness can be verified by the Rg. As seen in Fig. 5D, the Rg profile of the vaccine and TLR-4 complex initially decreased slightly after 5 ns before reaching a steady state. The overall peak of the Rg did not exhibit significant fluctuations, indicating a rigid state. The SASA value of the epitope and protein complex was initially lower, but it increased beyond the initial phase. The lower SASA value accounts for the protein's contracted nature [134], and the overall SASA peak signifies the robust nature of the complex. These data collectively suggest the stability of the TLR-4 vaccine complex, which could facilitate the transportation of the vaccine within the antigen-presenting cell. The PDBsum server reported four hydrogen bonds (A-C: 31Glu-249Thr, 32Val-211His, 35Asn-73Lys, 605Glu-126Thr) and 78 non-bonded interactions between TLR-4 (Chain A) and the vaccine (chain C). In contrast, two hydrogen bonds (B-C: 39Lys-219Pro, 143Glu-228Trp), one salt-bridge (B-C: 136Glu-227Arg), and 30 non-bonded interactions were reported between the MD molecule (Chain B) and the vaccine (chain C). All six hydrogen bonds were between 2-3 Å, indicating strong interactions between the vaccine-bound TLR-4/MD complex [135]. The inclusion of a salt-bridge interaction could enhance the biomolecular stability of the vaccine-TLR complex [136-138]. Hydrogen bond and salt-bridge interactions are shown in Table 4 and Fig. 6.

**Fig. 7.**
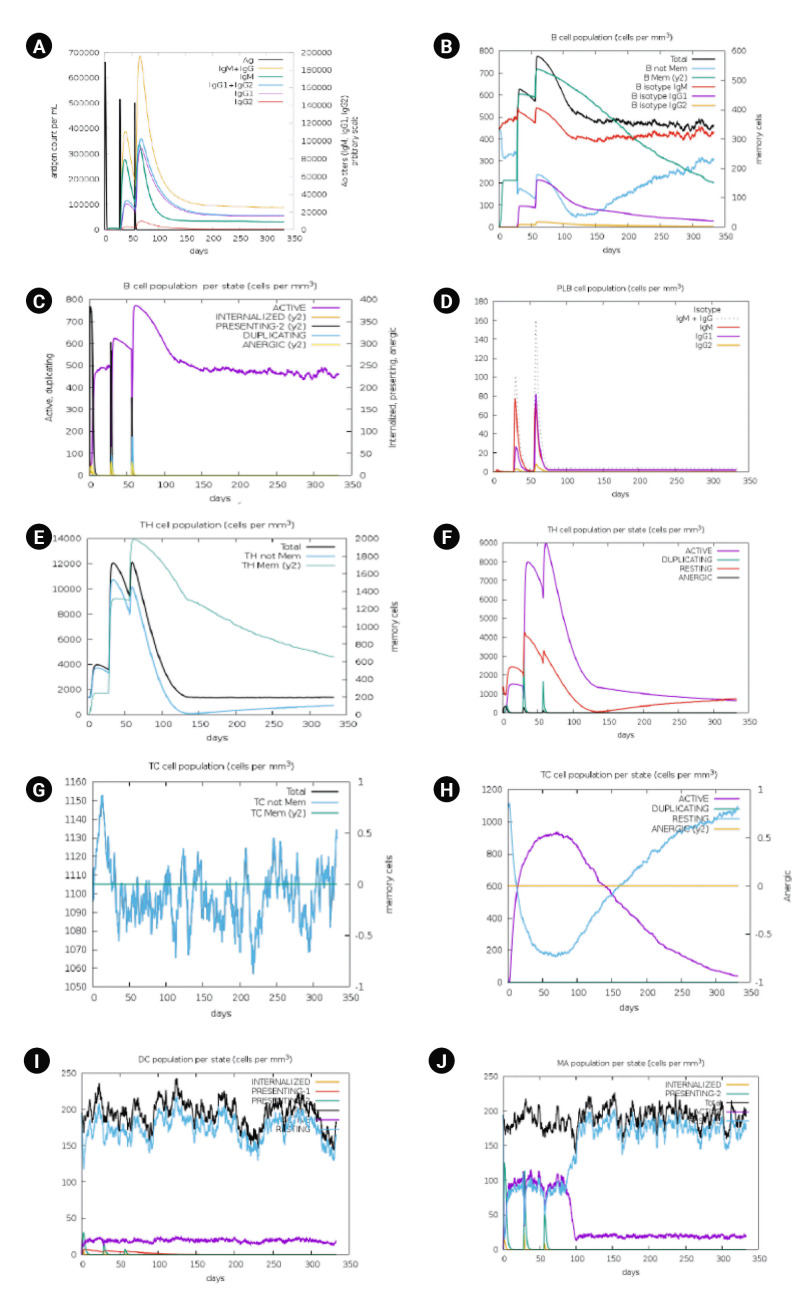
Immune simulation analysis of designed vaccine over the course of three injections. (A) Response of immunoglobulin and immunocomplex to vaccine inoculations. (B) Rise in B-cell population. (C) Inclination of B-cell population. (D) Rise in plasma B cell. (E) Enhancement of helper T cell. (F) Rise in helper T cell. (G) Increase in regulatory T lymphocyte. (H) Augmentation in cytotoxic T lymphocyte. (I) Rise in active dendritic cell. (J) Increase in macrophages.

Immune stimulation of the multi-epitope vaccine was conducted to determine if the epitopes and adjuvants could generate sufficient adaptive immunity. The analysis clearly demonstrated that the designed vaccine has the potential to trigger a typical immune response. A simulation involving three doses showed a subsequent increase in the primary immune response and stimulation of the secondary immune response. The vaccine was projected to significantly induce primary immune responses incrementally after each of the three injections (Fig. 7). Moreover, the secondary immune response was stimulated, and the primary immune response gradually increased after each dose (Fig. 7A). The number of B cells (Fig. 7B and 7C), plasma B cells (Fig. 7D), helper T cells (Fig. 7E and 7F), cytotoxic T cells (Fig. 7G and 7H) and antigen-presenting cells such as dendritic cells and macrophages (Fig. 7I and 7J) increased significantly after administration.

## Discussion

Tuberculosis, the leading cause of death from a single infectious agent, continues to confound thousands of scientists in this modern era [2]. The global prevalence of MDR and XDR Mtb strains can be attributed to the transmission of gene resistance through horizontal gene transfer and the acquisition of gene resistance via vertical gene transfer. The investigation of non-resistant gene products is crucial for the development of new antimicrobial targets and vaccine candidates. Vaccines have the potential to stimulate immune responses for decades without inducing resistance [139]. The human gut microflora is less likely to be impacted by vaccines compared to broad-spectrum antibiotics, which target multiple bacterial species simultaneously [140]. Traditional vaccine development is a time-consuming, laborious, and expensive process [35,141]. Several experimental studies have highlighted the immunoinformatics approach as a promising strategy for designing highly efficient, immunogenic, and safe vaccines [35].

Several drug repositioning studies have been conducted on druggable proteins of Mtb, in which multiple drugs were available either for selected proteins of Mtb or homologous proteins of other pathogenic species [78,121]. Two groups utilized subtractive proteomics to screen out promising therapeutic compounds [122,123], and two other groups screened out vaccine targets [77,124]. However, neither group conducted immunoinformatics to design a vaccine, nor did they predict MmpL4 as a potential drug target or vaccine candidate. One group carried out an immunoinformatics study, selecting 38 highly expressed Mtb proteins *in vivo* (in both humans and mice), but the study was limited to screening only B-cell and T-cell epitopes [142]. Although two other groups used immunoinformatics to design vaccines against Mtb, they did not use a subtractive proteomics approach to identify pathogen-specific, essential targets that are less homologous to gut microflora [9,143]. Several crucial steps of subtractive proteomics include host non-homology analysis, essential protein analysis, pathogen-specific pathway analysis, non-resistance analysis, subcellular localization analysis, and gut microbiome analysis.

In our study, we utilized a computational proteome subtraction approach to identify non-resistant, essential, antigenic membrane proteins. We predicted the transmembrane transport protein MmpL4 (NP_214964.1) as the most promising candidate for vaccine development. The MmpL4 protein was also predicted to be non-homologous to human anti-targets and showed limited similarity to gut microflora (less than 28% similarity with only five hits). The MmpL4 protein plays a crucial role in iron transportation. Mtb sustains its growth within the host by seizing and importing iron, a vital nutrient, into its cytoplasm. When cellular iron is scarce, the pathogen synthesizes siderophores (mycobactins and carboxymycobactins) to capture iron, thereby evading the host's immune system [51]. The MmpL4 protein, in conjunction with the MmpL5 protein, synthesizes and transports siderophores. The siderophore export accessory proteins MmpS4 and MmpS5, which are anchored in the membrane, facilitate this transportation. MmpL4 has been shown to interact with virulence factors (MmpS4 and MmpS5) (Fig. 2) [51]. Therefore, disrupting the function of the transmembrane transport protein MmpL4 and its interactions with other transporter proteins could result in iron deficiency in the pathogen, ultimately leading to the pathogen's death. The DrugBank Database results revealed no existing drug or homologous protein for MmpL4 (NP_214964.1), indicating the novelty of MmpL4 as a species-specific drug target. This protein is also predicted to be a potential antigenic protein and was found to be 99% conserved in 22 MDR and XRD strains of Mtb. Subsequently, we employed an immunoinformatics approach on the MmpL4 protein to design an efficient multi-epitope vaccine candidate. Therefore, our findings in this study have contributed advanced knowledge compared to previous studies

Antigenic epitopes, which include T-cell (CD8+ and CD4+) and B-cell epitopes, are crucial for designing an ideal multi-epitope vaccine and generating a specific immune response against predicted antigens [37]. The roles of CD8+ T cells and CD4+ T cells in responses against Mtb have been extensively reviewed by Prezzemolo et al. (2014) [144] and Lin et al. (2015) [145]. In short, it is well established that CD8+ T cells express perforin, granzyme, and granulysin, which induce apoptosis in Mtb-infected cells, such as macrophages, in both human and mouse models during antigen-specific responses [145-148]. Numerous studies have identified CD4+ T cells as crucial for controlling tuberculosis infections [149-151]. IFN-γ plays an important role in phagocytosis and oxidative bursts in *Mycobacterium* spp. [152]. Moreover, the activation of IFN-γ and tumor necrosis factor-α, mediated by Mtb-specific CD4+ T cells, recruits monocytes and granulocytes, thereby enhancing their antimicrobial activities [153-156]. CD4+ T cell-induced IFN-γ stimulates macrophages to synthesize nitric oxide, leading to the clearance of this pathogen [157-160]. Therefore, both CD8+ and CD4+ T-cell epitopes were screened from the MmpL4 protein, and the selection process was based on several critical properties such as antigenicity, immunogenicity, non-toxicity, non-allergenicity, external topology, and cytokine production [104]. Four CD8+ T-cell peptides and three CD4+ T-cell peptides containing multiple epitopes (clusters) were selected for the subunit vaccine design. Conversely, both naïve and memory B cells are found in the lungs of humans infected with tuberculosis [48], and they assist in antigen presentation to T cells, cytokine production, and Mtb-specific antibody generation [48]. Rao et al. (2015) [161] highlighted the role of B cells in the antibody-mediated response against tuberculosis. Kringelum et al. (2013) [162] reported that LBL epitopes are more stable than discontinuous epitopes, which is why we only screened LBL epitopes in our study. Five peptide regions containing LBL epitope clusters were selected for the vaccine design. The vaccine was assembled with adjuvants and linkers. The adjuvant 50s ribosomal protein L7/L12 of Mtb (130 amino acids), which has proven effective in stimulating TLR-4 [92], was used in the vaccine design, while the EAAAK linker enhances the stability and bi-functional catalytic activity of the fusion protein [163]. The PADRE sequence (AKFVAAWTLKAAA) was incorporated to address issues caused by highly polymorphic HLA alleles [164]. The invasin peptide (TAKSKKFPSYTATYQF) was used to enhance the immune response by the adenoviral DNA vaccine [165].

The designed vaccine was antigenic, non-toxic, highly stable, hydrophilic, soluble, and found to be stable as a vaccine-TLR4/MD docked complex through molecular dynamics simulation. Our group has computationally designed an adenoviral DNA vaccine for West Nile virus [33]. Adenoviral vaccines can be produced at a relatively low cost and at higher tiers of vaccines, making this platform widely studied and extensively evaluated for vaccine development [166-170]. Pharmaceutical companies such as Johnson & Johnson, AstraZeneca, CanSino Biologics Inc., and the Gamaleya Research Institute have recently marketed adenovirus-based DNA vaccines [171]. Therefore, our designed adenoviral DNA vaccine could be a potential candidate for Mtb control. Despite the promising results of the proposed vaccine, our study is based on computational subtractive proteomics and immunoinformatics approaches; we recommend laboratory validation to evaluate the efficacy of the proposed vaccine.

Experimental approaches can be time-consuming and laborious, often yielding minimal results. Consequently, bioinformatics approaches have become the preferred methods for scientists seeking to identify potential novel drug targets and vaccine candidates. This study utilized the subtractive proteomics approach to identify novel vaccine targets for 23 virulent Mtb strains. We ultimately selected a membrane protein, transmembrane transport protein MmpL4 NP_214964.1, as both a potential drug target and vaccine candidate. This protein, conserved in the other 22 virulent MDR and XDR strains of Mtb, demonstrated more than 99% sequence similarity. The pathogen-specific transmembrane transport protein MmpL4 is non-homologous to the host and essential for the pathogen's survival. MmpL4 proteins exhibited lower similarity with human gut microflora and were non-homologous to human anti-targets, thus being predicted as a novel therapeutic target. We further expanded our study to design a subunit vaccine from the MmpL4 protein using an immunoinformatics approach. The vaccine contained CTL, HTL, BCL epitopes, the 50s ribosomal protein L7/L12 of Mtb as a TLR-4 specific adjuvant, PADRE, and an invasin sequence to generate an appropriate immune response against tuberculosis infections. Therefore, this *in silico* study could save researchers both time and costs in finding effective solutions against infections caused by any of the 23 virulent MDR and XDR Mtb strains. This approach also reduces the need for extensive pre-clinical trials and repeated assay errors.

## Figures and Tables

**Table 1.** Subtractive proteomic analysis and its outcomes

No.	Steps	*Mycobacterium tuberculosis* H37Rv
1	Total number of proteins	3,906
2	Removal of paralogous sequences (>80%) and smaller sequences in CD-Hit	373
3	Essential proteins in DEG 15.2 server (E-value ≤ 10^–100^, bit score ≥ 100)	580
4	Human (host) non-homologous proteins of pathogen (E-value > 10^-4^)	330
5	Proteins involved in unique metabolic pathways	47
6	Novel targets passed through subcellular localization (CELLO, PSORTb, PSLPred), DrugBank, lower gut microflora non-homology (E-value 0.001, similarity <35%, hits <10), ExPASy/MW (<110 kDa)	5
7	Vaccine target with transmembrane helices	1

**Table 2.** Screening of 41 unique proteins based on localization, gut microflora non-homology, novelty, and molecular weight

No.	Accession No.	Subcellular location	Gut microflora similarity (hit < 10, similarity < 35%)	Druggability	Molecular weight (kDa)
1	NP_214599.1	Membrane	Passed	Novel	23.042
2	NP_214722.1	Cytoplasmic	Failed	Novel	28.546
3	NP_214865.1	Cytoplasmic	Failed	Novel	24.532
4	NP_214878.1	Membrane	Failed	Novel	24.489
5	NP_214916.1	Membrane	Passed	Novel	104.838
6	NP_214964.1	Membrane	Passed	Novel	105.234
7	NP_214979.1	Cytoplasmic	Failed	Novel	53.072
8	NP_215077.1	Membrane	Failed	Novel	30.681
9	NP_215156.1	Cytoplasmic	Failed	Druggable	34.670
10	NP_215157.1	Cytoplasmic	Failed	Druggable	33.262
11	NP_215172.1	Membrane	Failed	Novel	25.127
12	NP_215190.1	Membrane	Passed	Novel	104.785
13	NP_215271.1	Cytoplasmic	Failed	Druggable	27.513
14	NP_215368.1	Cytoplasmic	Failed	Druggable	59.783
15	NP_215418.1	Cytoplasmic	Failed	Druggable	25.253
16	NP_215543.1	Cytoplasmic	Failed	Druggable	24.741
17	NP_215686.1	Cytoplasmic	Passed	Novel	31.742
18	NP_215699.1	Membrane	Passed	Novel	106.415
19	NP_215737.1	Cytoplasmic	Failed	Novel	28.877
20	NP_215939.1	Cytoplasmic	Passed	Novel	34.933
21	NP_215973.1	Membrane	Failed	Novel	27.370
22	NP_216000.1	Cytoplasmic	Failed	Druggable	28.527
23	NP_216038.1	Membrane	Failed	Novel	122.430
24	NP_217050.1	Cytoplasmic	Failed	Novel	20.407
25	NP_217219.1	Cytoplasmic	Failed	Druggable	57.800
26	NP_217227.1	Cytoplasmic	Failed	Novel	25.232
27	NP_217236.2	Cytoplasmic	Failed	Novel	24.841
28	NP_217264.1	Unknown	Failed	Novel	94.406
29	NP_217272.1	Cytoplasmic	Failed	Novel	60.084
30	NP_217422.1	Cytoplasmic	Failed	Druggable	25.166
31	NP_217502.1	Cytoplasmic	Failed	Novel	22.187
32	NP_217763.1	Cytoplasmic	Failed	Druggable	25.279
33	NP_217895.1	Cytoplasmic	Passed	Novel	34.030
34	NP_217964.1	Cytoplasmic	Failed	Novel	131.896
35	NP_218175.1	Membrane	Failed	Novel	27.632
36	NP_218300.1	Membrane	Failed	Novel	32.336
37	NP_218323.1	Membrane	Failed	Novel	32.654
38	NP_218340.1	Membrane	Passed	Novel	115.998
39	NP_218403.1	Extracellular	Failed	Druggable	55.594
40	YP_177735.1	Membrane	Failed	Novel	26.488
41	YP_177866.1	Membrane	Failed	Novel	29.931

**Table 3.** Physiochemical properties, antigenicity, and allergenicity profiling of the constructed vaccine

Parameter	Value	Comment
Molecular weight (kDa)	47.581	Suitable
Theoretical PI	9.44	Basic in nature
Estimated half-life	30 h	Mammalian reticulocyte (*in vitro*)
	>20 h	Yeast (*in vivo*)
	>10 h	*Escherichia coli* (*in vivo*)
Instability index	36.14 (stable)	Stable
Aliphatic index	93.70	Thermostable
GRAVY	0.109	Slightly hydrophobic
VaxiJen score	0.7419	Antigenic
AlgPred	-	Non-allergic
SOLpro	0.902276	Highly soluble in *E. coli*

**Table 4.** Protein-protein interaction analysis after a 20-ns molecular dynamics simulation

Chain	Residues	Position	Bond type or Distance (Å)	Ligand	Residues	Position	Distance (Å)
Chain A (TLR-4)	Glu	31	Hydrogen	Chain C (vaccine)	Thr	249	2.67
	Val	32	Hydrogen		His	211	2.87
	Asn	35	Hydrogen		Lys	73	2.80
	Glu	605	Hydrogen		Thr	126	2.58
Chain B (MD)	Lys	39	Hydrogen	Chain C (vaccine)	Pro	219	2.75
	Glu	143	Hydrogen		Trp	228	2.86
	Glu	136	Salt bridge		Arg	227	2.94

TLR-4, Toll-like receptor-4; MD, molecular dynamics.

## Supplementary Materials

Supplementary Table 1. List of hypothetical proteins excluded in this study

Supplementary Table 2. Antigenicity score and number of transmembrane helices

Supplementary Table 3. List of 22 virulent strains of *Mycobacterium tuberculosis*

Supplementary Table 4. Screening of cytotoxic T lymphocyte (CTL) epitopes

Supplementary Table 5. Screening of helper T lymphocyte (HTL) epitopes

Supplementary Table 6. Screening of B-cell lymphocyte (BCL) epitopes

Supplementary Table 7. Quality validation of Three Dimensional (3D) structure of constructed vaccine and refined structures

Supplementary File 1. List of 83 Gut Microflora strains.

Supplementary File 2. List of 95 Gut Microflora strains.

Supplementary File 3. List of accession numbers of 210 Anti-target proteins.
