# Supplementary material for: Multi-epitope vaccine against drug-resistant strains of Mycobacterium tuberculosis: a proteome-wide subtraction and immunoinformatics approach
Source: Genomics Inform. 2023 Sep 27;21(3):e42. doi: 10.5808/gi.23021 (PMC10584640; doi:10.5808/gi.23021)
Supplement: Supplementary Table 1. — List of hypothetical proteins excluded in this study [file gi-23021-Supplementary-Table-1.pdf]

Supplementary Table S1. List of hypothetical proteins excluded in this study

S. No.	Accession No.	KO assignment
1	NP_214886.1	KO7402
2	NP_215054.1	KO9931
3	NP_215188.1	KO2616
4	NP_215937.1	KO6958
5	NP_216934.1	KO2797
6	NP_217442.1	KO7040

KO, KEGG Ontology.
