# Supplementary material for: Multi-epitope vaccine against drug-resistant strains of Mycobacterium tuberculosis: a proteome-wide subtraction and immunoinformatics approach
Source: Genomics Inform. 2023 Sep 27;21(3):e42. doi: 10.5808/gi.23021 (PMC10584640; doi:10.5808/gi.23021)
Supplement: Supplementary Table 2. — Antigenicity score and number of transmembrane helices [file gi-23021-Supplementary-Table-2.pdf]

Supplementary Table 2. Antigenicity score and number of transmembrane helices

S. No.	Accession No.	VaxiJen score	TMHMM	Subcellular location
1	NP_214599.1	0.3357	7	Membrane
2	NP_214916.1	0.3983	12	Membrane
**3**	**NP_214964.1**	**0.4165**	**11**	**Membrane**
4	NP_215190.1	0.3810	12	Membrane
5	NP_215699.1	0.3606	11	Membrane
