# Supplementary material for: Multi-epitope vaccine against drug-resistant strains of Mycobacterium tuberculosis: a proteome-wide subtraction and immunoinformatics approach
Source: Genomics Inform. 2023 Sep 27;21(3):e42. doi: 10.5808/gi.23021 (PMC10584640; doi:10.5808/gi.23021)
Supplement: Supplementary Table 3. — List of 22 virulent strains of Mycobacterium tuberculosis [file gi-23021-Supplementary-Table-3.pdf]

Supplementary Table 3. List of 22 virulent strains of *Mycobacterium tuberculosis*

S. No.	Strain
1	*Mycobacterium tuberculosis* CDC1551
2	*Mycobacterium tuberculosis* H37RA
3	*Mycobacterium tuberculosis* F11
4	*Mycobacterium tuberculosis* KZN 1435
5	Mycobacterium tuberculosis str. Haarlem
6	*Mycobacterium tuberculosis* KZN 4207
7	*Mycobacterium tuberculosis* KZN 605
8	*Mycobacterium tuberculosis* CTRI-2
9	*Mycobacterium tuberculosis* CCDC5079
10	*Mycobacterium tuberculosis* CCDC5180
11	*Mycobacterium tuberculosis* 7199-99
12	*Mycobacterium tuberculosis* strain Erdman
13	*Mycobacterium tuberculosis* strain BEIJING/NITR203
14	*Mycobacterium tuberculosis* EAI5-NITR206
15	*Mycobacterium tuberculosis* EAI5
16	*Mycobacterium tuberculosis* HKBS1
17	*Mycobacterium tuberculosis* BT2
18	*Mycobacterium tuberculosis* BT1
19	*Mycobacterium tuberculosis* K
20	*Mycobacterium tuberculosis* 49-02
21	*Mycobacterium tuberculosis* H37RVSIENA
22	*Mycobacterium tuberculosis* str. Kurono
