# Supplementary material for: Multi-epitope vaccine against drug-resistant strains of Mycobacterium tuberculosis: a proteome-wide subtraction and immunoinformatics approach
Source: Genomics Inform. 2023 Sep 27;21(3):e42. doi: 10.5808/gi.23021 (PMC10584640; doi:10.5808/gi.23021)
Supplement: Supplementary Table 7. — Quality validation of Three Dimensional (3D) structure of constructed vaccine and refined structures [file gi-23021-Supplementary-Table-7.pdf]

Supplementary Table 7. Quality validation of the three-dimensional (3D) structure of the constructed vaccine and refined structures

Model	GDT-HA	RMSD	MolProbity	Clash score	Poor rotamers	Rama favored
Initial	1	0	1.484	2.9	0.3	93.9
MODEL 1	0.9853	0.295	2.055	19.9	0.6	96.1
MODEL 2	0.9774	0.319	2.034	18.8	0.3	96.1
MODEL 3	0.9831	0.312	2.117	20.3	0.3	95.5
MODEL 4	0.9797	0.318	2.089	20.6	0.6	95.9
MODEL 5	0.9814	0.311	2.092	19.9	0.3	95.7

RMSD, root mean square deviation.
