# Supplementary material for: Multi-epitope vaccine against drug-resistant strains of Mycobacterium tuberculosis: a proteome-wide subtraction and immunoinformatics approach
Source: Genomics Inform. 2023 Sep 27;21(3):e42. doi: 10.5808/gi.23021 (PMC10584640; doi:10.5808/gi.23021)
Supplement: Supplementary File 1. — List of 83 Gut Microflora strains. [file gi-23021-Supplementary-File-1.docx]

**Supplementary File 1.** List of 83 gut microflora strains

Actinomyces_odontolyticus_ATCC_17982

Akkermansia_muciniphila_ATCC_BAA-835

Alistipes_putredinis_DSM_17216

Anaerofustis_stercorihominis_DSM_17244

Anaerostipes_caccae_DSM_14662

Anaerotruncus_colihominis_DSM_17241

Pseudoflavonifractor_capillosus_ATCC_29799

Bacteroides_cellulosilyticus_DSM_14838

Bacteroides_coprocola_DSM_17136

Bacteroides_dorei_DSM_17855

Bacteroides_eggerthii_DSM_20697

Bacteroides_finegoldii_DSM_17565

Bacteroides_intestinalis_DSM_17393

Bacteroides_ovatus_ATCC_8483

Bacteroides_pectinophilus_ATCC_43243

Bacteroides_plebeius_DSM_17135

Bacteroides_stercoris_ATCC_43183

Bacteroides_uniformis_ATCC_8492

Bifidobacterium_adolescentis_ATCC_15703

Bifidobacterium_adolescentis_L2-32

Bifidobacterium_angulatum_DSM_20098

Bifidobacterium_bifidum_DSM_20456

Bifidobacterium_breve_DSM_20213

Bifidobacterium_dentium_ATCC_27678

Bifidobacterium_longum_DJO10A

Bifidobacterium_longum_NCC2705

Bifidobacterium_longum_subsp_infantis_str_ATCC_15697

Borrelia_burgdorferi_CA-11.2A

Butyrivibrio_crossotus_DSM_2876

Catenibacterium_mitsuokai_DSM_15897

Clostridium_asparagiforme_DSM_15981

Clostridium_bartlettii_DSM_16795

Clostridium_bolteae_ATCC_BAA-613

Clostridium_hiranonis_DSM_13275

Clostridium_leptum_DSM_753

Clostridium_methylpentosum_DSM_5476

Clostridium_nexile_DSM_1787

Clostridium_ramosum_DSM_1402

Clostridium_scindens_ATCC_35704

Clostridium_sp_L2-50

Clostridium_sp_M62/1

Clostridium_sp_SS2/1

Clostridium_spiroforme_DSM_1552

Clostridium_sporogenes_ATCC_15579

Clostridium_symbiosum_ATCC_14940

Collinsella_aerofaciens_ATCC_25986

Collinsella_intestinalis_DSM_13280

Collinsella_stercoris_DSM_13279

Coprococcus_comes_ATCC_27758

Coprococcus_eutactus_ATCC_27759

Dorea_formicigenerans_ATCC_27755

Dorea_longicatena_DSM_13814

Eggerthella_lenta_DSM_2243

Enterobacter_cancerogenus_ATCC_35316

Eubacterium_dolichum_DSM_3991

Eubacterium_hallii_DSM_3353

Eubacterium_siraeum_DSM_15702

Eubacterium_ventriosum_ATCC_27560

Faecalibacterium_prausnitzii_A2-165

Faecalibacterium_prausnitzii_M21/2

Lactobacillus_salivarius_UCC118

Methanobrevibacter_smithii_ATCC_35061

Methanobrevibacter_smithii_DSM_11975

Methanobrevibacter_smithii_DSM_2374

Methanobrevibacter_smithii_DSM_2375

Mitsuokella_multacida_DSM_20544

Parabacteroides_johnsonii

Parabacteroides_merdae_ATCC_43184

Parvimonas_micra_ATCC_33270

Photorhabdus_luminescens_subsp_laumondii_TTO1

Prevotella_copri_DSM_18205

Providencia_alcalifaciens_DSM_30120

Providencia_rettgeri_DSM_1131

Providencia_rustigianii_DSM_4541

Roseburia_faecis_M72/1

Roseburia_intestinalis_L1-82

Ruminococcus_gnavus_ATCC_29149

Ruminococcus_lactaris_ATCC_29176

Ruminococcus_obeum_ATCC_29174

Ruminococcus_torques_ATCC_27756

Streptococcus_infantarius_subsp_infantarius_ATCC_BAA-102

Subdoligranulum_variabile_DSM_15176

Victivallis_vadensis_ATCC_BAA-548
