# Supplementary material for: Multi-epitope vaccine against drug-resistant strains of Mycobacterium tuberculosis: a proteome-wide subtraction and immunoinformatics approach
Source: Genomics Inform. 2023 Sep 27;21(3):e42. doi: 10.5808/gi.23021 (PMC10584640; doi:10.5808/gi.23021)
Supplement: Supplementary File 2. — List of 95 Gut Microflora strains. [file gi-23021-Supplementary-File-2.docx]

**Supplementary File 2.** List of 95 gut microflora strains

1. Actinomyces odontolyticus ATCC 17982

2. Akkermansia muciniphila ATCC BAA-835

3. Alistipes putredinis DSM 17216

4. Anaerofustis stercorihominis DSM 17244

5. Anaerostipes caccae DSM 14662

6. Anaerotruncus colihominis DSM 17241

7. Bacteroides capillosus ATCC 29799

8. Bacteroides cellulosilyticus DSM 14838

9. Bacteroides coprocola DSM 17136

10. Bacteroides dorei DSM 17855

11. Bacteroides eggerthii DSM 20697

12. Bacteroides finegoldii DSM 17565

13. Bacteroides intestinalis DSM 17393

14. Bacteroides ovatus ATCC 8483

15. Bacteroides pectinophilus ATCC 43243

16. Bacteroides plebeius DSM 17135

17. Bacteroides stercoris ATCC 43183

18. Bacteroides uniformis ATCC 8492

19. Bifidobacterium adolescentis ATCC 15703

20. Bifidobacterium adolescentis L2-32

21. Bifidobacterium angulatum DSM 20098

22. Bifidobacterium bifidum DSM 20456

23. Bifidobacterium breve DSM 20213

24. Bifidobacterium dentium ATCC 27678

25. Bifidobacterium longum BORI

26. Bifidobacterium longum DJO10A

27. Bifidobacterium longum DJO10A

28. Bifidobacterium longum NCC2705

29. Bifidobacterium longum subsp. infantis str. ATCC 15697

30. Borrelia burgdorferi CA-11.2a

31. Bryantella formatexigens DSM 14469

32. Butyrivibrio crossotus DSM 2876

33. Catenibacterium mitsuokai DSM 15897

34. Citrobacter sp. ATCC 29220

35. Clostridium asparagiforme DSM 15981

36. Clostridium bartlettii DSM 16795

37. Clostridium bolteae ATCC BAA-613

38. Clostridium hiranonis DSM 13275

39. Clostridium leptum DSM 753

40. Clostridium methylpentosum DSM 5476

41. Clostridium nexile DSM 1787

42. Clostridium ramosum DSM 1402

43. Clostridium scindens ATCC 35704

44. Clostridium sp. A2-232

45. Clostridium sp. L2-50

46. Clostridium sp. M62/1

47. Clostridium sp. SS2/1

48. Clostridium spiroforme DSM 1552

49. Clostridium sporogenes ATCC 15579

50. Clostridium symbiosum ATCC 14940

51. Collinsella aerofaciens ATCC 25986

52. Collinsella intestinalis DSM 13280

53. Collinsella stercoris DSM 13279

54. Coprococcus comes ATCC 27758

55. Coprococcus eutactus ATCC 27759

56. Desulfitobacterium hafniense DSM 13498

57. Dorea formicigenerans ATCC 27755

58. Dorea longicatena DSM 13814

59. Eggerthella lenta DSM 2243

60. Enterobacter cancerogenus ATCC 35316

61. Eubacterium dolichum DSM 3991

62. Eubacterium hallii DSM 3353

63. Eubacterium siraeum DSM 15702

64. Eubacterium ventriosum ATCC 27560

65. Faecalibacterium prausnitzii A2-165

66. Faecalibacterium prausnitzii M21/2

67. Lactobacillus reuteri F275

68. Lactobacillus reuteri F275

69. Lactobacillus salivarius UCC118

70. Methanobrevibacter smithii ATCC 35061

71. Methanobrevibacter smithii DSM 11975

72. Methanobrevibacter smithii DSM 2374

73. Methanobrevibacter smithii DSM 2375

74. Mitsuokella multacida DSM 20544

75. Parabacteroides johnsonii

76. Parabacteroides merdae ATCC 43184

77. Parvimonas micra ATCC 33270

78. Photorhabdus luminescens subsp. laumondii TTO1

79. Prevotella copri DSM 18205

80. Providencia alcalifaciens DSM 30120

81. Providencia rettgeri DSM 1131

82. Providencia rustigianii DSM 4541

83. Roseburia faecis M72/1

84. Roseburia intestinalis L1-82

85. Ruminococcus callidus ATCC 27760

86. Ruminococcus gnavus ATCC 29149

87. Ruminococcus hansenii DSM 20583

88. Ruminococcus hydrogenotrophicus DSM 10507

89. Ruminococcus lactaris ATCC 29176

90. Ruminococcus obeum ATCC 29174

91. Ruminococcus sp. GM2/1

92. Ruminococcus torques ATCC 27756

93. Streptococcus infantarius subsp. infantarius ATCC BAA-102

94. Subdoligranulum variabile DSM 15176

95. Victivallis vadensis ATCC BAA-548
